# Peptides against Low Density Lipoprotein (LDL) Aggregation Inhibit Intracellular Cholesteryl Ester Loading and Proliferation of Pancreatic Tumor Cells

**DOI:** 10.3390/cancers14040890

**Published:** 2022-02-11

**Authors:** Aleyda Benitez-Amaro, Neus Martínez-Bosch, Noemí Manero-Rupérez, Lene Claudi, Maria Teresa La Chica Lhoëst, Marta Soler, Lia Ros-Blanco, Pilar Navarro, Vicenta Llorente-Cortés

**Affiliations:** 1 Institute of Biomedical Research of Barcelona (IIBB), Spanish National Research Council (CSIC), 08036 Barcelona, Spain; abenitez@santpau.cat (A.B.-A.); lclaudi@santpau.cat (L.C.); mlachica@santpau.cat (M.T.L.C.L.); 2 Biomedical Research Institute Sant Pau (IIB Sant Pau), 08025 Barcelona, Spain; msoler@santpau.cat (M.S.); lros@santpau.cat (L.R.-B.); 3 Cancer Research Program, Hospital del Mar Medical Research Institute (IMIM), Unidad Asociada IIBB-CSIC, 08003 Barcelona, Spain; nmartinez@imim.es (N.M.-B.); nmanero@imim.es (N.M.-R.); 4 August Pi Sunyer Biomedical Research Institute (IDIBAPS), 08036 Barcelona, Spain; 5 CIBERCV, Institute of Health Carlos III, 28029 Madrid, Spain

**Keywords:** anti-LDL aggregation peptides, LDL atherogenicity, cholesteryl esters, pancreatic ductal adenocarcinoma

## Abstract

**Simple Summary:**

Dyslipidemia is a modifiable risk factor for pancreatic ductal adenocarcinoma (PDAC), one of the most lethal cancers. A key component of dyslipidemia is a high level of small and dense low-density lipoproteins (LDLs). These LDLs have a high probability to be entrapped and modified (aggregated) in the extracellular matrix (ECM), becoming a source of cholesterol for tumor cells. However, the effect of aggregated LDLs on tumor progression has been unexplored. The aim of this work was to determine the effect of modified LDLs on intracellular cholesteryl ester/free cholesterol ratio (CE/FC) and cancer cell growth, and the efficacy of peptides designed to inhibit LDL aggregation on these processes. Our results show that aggregated LDL upregulates the intracellular CE/FC ratio and cell growth in pancreatic cancer and that these upregulatory effects were blocked by peptides against LDL aggregation. We propose that anti-LDL aggregation peptides deserve to be further investigated as anti-tumoral strategies.

**Abstract:**

Dyslipidemia, metabolic disorders and/or obesity are postulated as risk factors for pancreatic ductal adenocarcinoma (PDAC). The majority of patients with these metabolic alterations have low density lipoproteins (LDLs) with increased susceptibility to become aggregated in the extracellular matrix (ECM). LDL aggregation can be efficiently inhibited by low-density lipoprotein receptor-related protein 1 (LRP1)-based peptides. The objectives of this work were: (i) to determine if aggregated LDLs affect the intracellular cholesteryl ester (CE)/free cholesterol (FC) ratio and/or the tumor pancreatic cell proliferation, using sphingomyelinase-modified LDL particles (Aggregated LDL, AgLDL); and (ii) to test whether LRP1-based peptides, highly efficient against LDL aggregation, can interfere in these processes. For this, we exposed human pancreatic cancer cell lines (PANC-1, RWP-1 and Capan-1) to native (nLDL) or AgLDLs in the absence or presence of LRP1-based peptides (DP3) or irrelevant peptides (IP321). Results of thin-layer chromatography (TLC) following lipid extraction indicate that AgLDLs induce a higher intracellular CE/FC ratio than nLDL, and that DP3 but not IP321 counteracts this effect. AgLDLs also increase PANC-1 cell proliferation, which is inhibited by the DP3 peptide. Our results indicate that AgLDL-induced intracellular CE accumulation plays a crucial role in the proliferation of pancreatic tumor cell lines. Peptides with anti-LDL aggregation properties may thus exhibit anti-tumor effects.

## 1. Introduction

Pancreatic ductal adenocarcinoma (PDAC) presents one of the toughest challenges in oncology. Patients diagnosed with local and regional disease show a five-year relative survival rate of about 9% [1], which places PDAC as the third leading cause of cancer-related death in developed countries. Many advances in PDAC biology have been made [2,3]; however, to change the dismal prognosis of PDAC, improvements must be made by addressing early diagnosis and improving the ineffective therapies that are currently available.

Dyslipidemia, metabolic disorders and/or obesity have been postulated to be modifiable risk factors for PDAC [4,5,6]. People with metabolic disorders including obesity and type 2 diabetes have increased numbers of small dense lipoproteins that, in addition to high triglycerides and low high-density lipoprotein (HDL), conform the characteristic lipid profile of atherogenic dyslipemia [7]. Small dense lipoproteins are prone to binding to the extracellular matrix (ECM)-proteoglycans (PGs) [8,9]. Low-density lipoproteins (LDLs) play a crucial role as a source of exogenous cholesterol for cells, especially when LDLs become entrapped, as shown in the intimal [10] and proposed in the stromal [11] ECM of atherosclerotic plaques and tumors, respectively. Modulation of stroma-tumor crosstalk is essential for targeting PDAC progression [12,13]. In particular, pancreatic tumor stroma contains abundant ECM proteins, endothelial cells, immune cells and cancer-associated fibroblasts (CAFs) that represent up to 80% of the pancreatic tumor. In the atherosclerosis arena, the proatherogenic nature of LDLs is determined by the tendency of LDLs to be entrapped in the network of intimal PGs [8,9] and become aggregated [10,14]. In the ECM of human atherosclerotic lesions, sphingomyelinase (SMase) is one of the main secretory phospholipases contributing to aggregation of intimal retained LDL [15]. These events can also contribute to cancer progression, like in metastatic melanoma, where SMase has been reported to be activated by acidic extracellular pH [16], or in PDAC, where there is an initial and progressive alteration of the ECM [17] that may render PGs more interactive with lipoproteins.

In the vasculature, PGs promote the formation of aggregated LDLs (AgLDLs), which are taken up through the low-density lipoprotein receptor-related protein 1 (LRP1), a receptor that is upregulated by cholesterol and hypoxia in ECM-adjacent cells. Overall, this generates a vicious cycle that efficiently produces intracellular lipid droplets (LDs) and prothrombotic cell phenotypes in the vasculature [18,19,20]. In addition, increased cholesterol loading and changes in lipid metabolism due to vascular cells from the stroma can also modulate tumor angiogenesis [21,22], which in turn can contribute to the strong immunosuppressive microenvironment of PDAC [23].

In pancreatic cancer, lipid droplets (which closely correlate with tumor aggressiveness) are mainly formed by cholesteryl esters (CEs) from exogenous sources [24,25]. Moreover, PDAC tumors are enriched in activated pathways associated with lipoprotein-related metabolism [26], and an aberrant accumulation of CEs has been observed in human pancreatic cancer tissues and linked to cancer metastasis [27].

The sequence (Gly^1127^–Cys^1140^) (P3) located in the CR9 domain of cluster II of the LRP1 α-chain interacts with the apolipoprotein B100 (ApoB100) of modified lipoproteins [28]. Peptides based on this P3 sequence electrostatically interact with ApoB100 and maintain ApoB100 conformation, preserving LDL against aggregation induced by phospholipolytic enzymes present in the ECM [29,30]. In the vascular scenario, innovative therapeutic attempts, such as peptides anti-LDL aggregation [29,30,31,32] and their antibodies [33,34,35], have successfully inhibited the formation of lipid droplet cell phenotypes and atherosclerosis. The retro-enantio (e.g., reversed d-amino acid order) peptide of P3, termed DP3 (Ac-dN·dE·dE·dD·dS·dN·dD·dE·dS·dD·dN·dD·G-NH_2_) and an ApoB-100 based peptide, termed IP321 (H-RLTRKRGLK-NH_2_), have been previously validated by our group as positive and negative controls, respectively, in biochemical and cell assays developed to test the anti-LDL aggregation and anti-cholesteryl ester loading properties of a new family of optimized peptides [30].

Currently, it is unknown whether LDL aggregation determines the intracellular CE content and growth of pancreatic tumor cells. Of note, the ECM of the stroma in tumors, like that of the intima in atherosclerosis, contains PGs and lipolytic enzymes that may strongly facilitate LDL retention and aggregation. Our hypothesis is that aggregated LDLs provide a relatively inexhaustible source of cholesterol for PDAC cells and are a contributing factor for cell growth. Our objectives were: (1) to analyze the impact of aggregated LDLs on intracellular CE accumulation and proliferation of human pancreatic tumor cells; (2) to analyze the effect of peptides against LDL aggregation on intracellular CE accumulation and tumor cell proliferation, and (3) to know whether the effect of aggregated LDLs on cell growth depends on their capacity to transfer CEs to tumor cells.

## 2. Materials and Methods

### 2.1. Peptide Sequence and Synthesis

Peptide sequences were written from N to C, H- at the end terminus indicating amine ending, whereas -NH_2_ at the C terminus indicates amide. The amino acid sequence of LP3 peptide (parent) is H-GDNDSEDNSDEENC-NH_2_, DP3 is the retro-enantio version of LP3 without cysteine: H-needsndesdndh-NH_2_, where the amino acid letter code in lowercase stands for amino acids with D-chirality. As a negative control we used an ApoB-100-based peptide coded as IP321 with the sequence H-RLTRKRGLK-NH_2_. All peptides were synthesized by Iproteos (Barcelona Knowledge Campus, Barcelona, Spain) by standard 9-fluorenylmethoxycarbonyl/tert-butyl (Fmoc/tBu) solid-phase peptide synthesis. Syntheses were performed manually on a 100 μmol scale/each using Rink amide ChemMatrix^®^ resin. Peptide elongation and other solid-phase manipulations were done manually in polypropylene syringes, each fitted with a polyethylene porous disk. Solvents and soluble reagents were removed by suction. Washings between synthetic steps were done with dimethylformamide and dichloromethane using 5 mL of solvents/g resin each time. Nα-Fmoc-protected amino acids (four equivalents) were coupled using 2-(1H-benzotriazole-1-yl)-1,1,3,3-tetramethyluronium (TBTU, four equivalents), and N,N-diisopropylethylamine (eight equivalents). The extent of the reaction was monitored using the Kaiser test (primary amines). During couplings, the mixture was allowed to react with intermittent manual stirring. The Fmoc group was removed by treating the resin with 20% piperidine in (N,N-dimetilformamide) DMF (3 mL/g resin). The peptides were cleaved from the resin using a mixture of trifluoroacetic acid, triisopropylsilane and water (95:2.5:2.5). The crude products obtained were purified in reverse phase using a semiprep HPLC instrument equipped with a C18 column. The purity and identity of the synthesized peptides was assessed by HPLC, HPLC-MS and MALDI-TOF analysis.

### 2.2. LDL Isolation and Purification

Human LDL (d1.019–d1.063 g/mL) was obtained from pooled normolipemic human plasma by sequential ultracentrifugation in a KBr density gradient. Briefly, very low density lipoproteins (VLDLs) were first discarded after spinning plasma at 36,000 rpm for 18 h at 4 °C using a fixed-angle rotor (50.2 Ti, Beckman Coulter, Brea, CA, USA) mounted on an Optima L-100 XP ultracentrifuge (Beckman Coulter). Subsequently, VLDL-free plasma was layered with 1.063 g/mL KBr solution and centrifuged at 36,000 rpm for 18 h at 4 °C. LDLs were dialyzed against 0.02 M Trizma, 0.15 M NaCl, 1 mM EDTA, pH 7.5 for 18 h, and then against normal saline for 2 h. Finally, isolated LDLs were filter-sterilized (0.22 μm Millex-GV filter unit, Merck KGaA, Darmstadt, Germany,). Protein concentration was determined using the BCA protein assay (ThermoFisher Scientific, Waltham, MA, USA) and the cholesterol concentration with a commercial kit (IL test Cholesterol, Izasa Scientific, Alcobendas, Spain).

### 2.3. LDL Modification by Sphingomyelinase (SMase) in the Presence and Absence of Peptides

To generate aggregated LDL (AgLDL), LDL (1.44 mg/mL) were incubated with 40 U/L of Bacillus cereus SMase (Sigma-Aldrich, Schnelldorf, Germany) in 20 mM Tris buffer (pH 7.0) containing 150 mM NaCl, 2 mM CaCl_2_, and 2 mM MgCl_2_ at 37 °C. From the methodological point of view, the in vitro modification of LDL by SMase has the main advantage versus other methods such as mechanical aggregation, in that the enzymatic modification is easily monitored and controlled. Therefore, the results using this kind of LDL modification are highly reproducible in terms of LDL aggregation degree (monitored by turbidimetry). LDL incubation with SMase was performed at several peptide concentrations ranging from 0 μM to 10 μM (0, 2.5, 5, 7.7, 8.8 and 10 μM). LDL lipolysis was stopped by the addition of EDTA (final concentration 0.5 mM).

### 2.4. Characterization of SMase-Induced LDL Aggregation by Turbidimetry

The LDL composition was measured by commercial methods adapted to a Cobas 6000/c501 autoanalyzer (Roche Diagnostics, Indianapolis, IN, USA). Total cholesterol, triglycerides and ApoB reagents were obtained from Roche Diagnostics. PLs, NEFAs, and free cholesterol reagents were obtained from Wako Pure Chemicals (Tokyo, Japan). Sample turbidity (LDL aggregation monitorization) was determined by measuring absorbance of 100 μL of LDLs (1.44 mg/mL ApoB) in a 96-well microplates at 405 nm.

### 2.5. Cell Culture of Pancreatic Tumor Cell Lines 

Human pancreatic cancer cell lines PANC-1 [36], RWP-1 [37], and Capan-1 [38] were obtained from the cancer cell repository at Hospital del Mar Medical Research Institute (IMIM), Barcelona. Cells were cultured at 37 °C in a cell incubator at 5% CO_2_ using DMEM (Invitrogen, Waltham, MA, USA) with 10% FBS.

### 2.6. Monitorization of the Efficacy of Peptides to Inhibit LDL-Induced Intracellular Cholesteryl Ester Accumulation

Peptide inhibitory efficacy was monitored by determination of intracellular cholesteryl ester/free cholesterol (CE/FC) ratio. PANC-1, RWP-1 and Capan-1 were seeded in 12-well plates (20,000 cells). After 24 h, cell quiescence was induced for 24 h with 0.2% fetal calf serum. Quiescent cells were exposed for different times to LDL previously treated with SMase for 18 h in the presence or absence of peptides at a concentration of 10 µM (ratio peptide/apoB100 5:1). The degree of LDL aggregation was assessed by turbidimetry measurements before the incubation with the cells. Following the lipoprotein incubation period, all cell types were washed exhaustively: twice with phosphate buffered saline (PBS), twice with PBS supplemented with 1% bovine serum albumin (BSA), and once with PBS supplemented with both 1% BSA and 100 U/mL heparin. Cells were then harvested into 1 mL of 0.15 M NaOH for lipid extraction followed by neutral intracellular lipid partitioning through thin layer chromatography and lipid band analysis and quantification.

### 2.7. Determination of Intracellular Cholesteryl Ester/Free Cholesterol Ratio

Lipids were extracted using the Bligh and Dyer method with minor modifications [24]. The lipid extract was dissolved in dichloromethane, applied to silica gel plates, and separated by thin layer chromatography. Cholesterol and cholesterol palmitate were run as standards of free and cholesteryl ester, respectively. A primary solvent combination of heptane/diethyl ether/acetic acid (74:21:4, *v*/*v*/*v*) was used as a chromatographic mobile phase followed by heptane alone. After lipid separation, the plates were dried and stained as previously reported [24]. Finally, the spots corresponding to CE and FC were measured by densitometry against the standard curve using a GS-800 Calibrated Densitometer (Bio-Rad, Hercules, CA, USA). Intracellular CEs detected in these cells upon exposure to LDL derives exclusively from CE supplied by LDL, as cells unexposed to LDL did not have intracellular CEs. The inhibitory effect of the peptide on the intracellular cholesterol accumulation was analyzed in terms of the decrease in the ratio of intracellular CE and FC content.

### 2.8. Confocal Microscopy Analysis

PANC-1, RWP-1, Capan-1 were seeded at 50,000 cells over glass coverslips in 24-well plates. Cells were left with 0.2% FBS for 24 h and exposed for 4 h to nLDL or AgLDL (100 µg/mL), in the absence or presence of DP3 or IP321 peptides (10 µM). Cells were fixed with PFA 4 % and permeabilized with 0.25 % Triton X-100. After blocking with 1% BSA, cells were incubated with BODIPY^®^ 493/503 (stock 5 mg/mL, dilution 1/100, ThermoFisher Scientific, Waltham, MA, USA). Hoechst 33342 (stock 10 mg/mL, dilution 1/2000) staining was used to counter visualize nucleus. Confocal images were acquired with Axio-Observer Z1 (ZEISS, Oberkochen, Germany) laser confocal microscope adapted to for sequential image acquisition. To assure a correct normalization of fluorescence, we have adjusted exposure times to eliminate the autofluorescence of the negative control (PANC-1 cells unexposed to LDL).

### 2.9. Flow Cytometry Analysis

Cells were grown in media supplemented with 10 % FBS for 24 h and arrested for 24 h in media supplemented with 0.2% FBS before LDL addition (time 0). At time 0, cells were exposed to LDL (added to the media without serum) for 24, 48 or 72 h. The experimental setting was designed to avoid serum interferences and to assure that cholesterol-induced cell growth was a direct response to cholesterol from LDL. Quiescent human pancreatic cancer cells, PANC-1, were exposed to nLDL or AgLDL (1.44 mg/mL) untreated or treated with peptide DP3 or IP321 (10 µM). Cells were then washed twice with PBS and removed from the culture dish with Trypsin-EDTA 0.25% (Invitrogen, Waltham, MA, USA). PBS supplemented with 1% FBS was used to inactivate the enzyme and harvest the cells. The cell suspension was centrifuged at 1200 rpm for 10 min. The resulting pellet was resuspended in PBS supplemented with propidium iodide (stock 1 mg/mL, dilution 1/1000) to detect dead cells. Live, dead and total cells were counted using a MACSQuant^®^ Analyzer with MACSQuantify™ Software version 2.13 (Miltenyi Biotec, Bergisch Gladbach, Germany).

### 2.10. Statistical Analysis

Data were described as the mean ± SEM. Experiments were performed three times and included three technical replicates unless otherwise indicated. Comparisons among groups were performed by parametric student *t*-test (one-factor analysis of variance). Statistical significance was considered when *p* was <0.05. The statistical software package SPSS (V25, IBM, Armonk, NY, USA) was used for statistical analyses.

## 3. Results

### 3.1. Effect of SMase on Human LDL Aggregation

**Figure 1:**
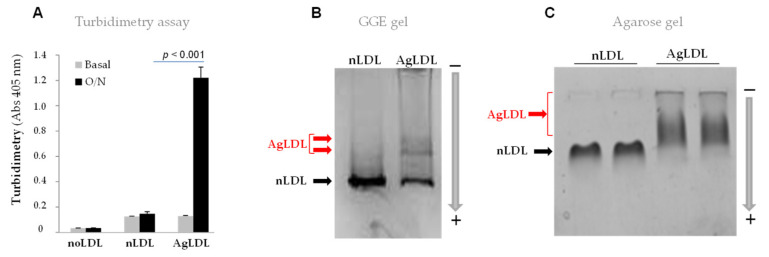
Effects of SMase on human LDL aggregation. (**A**) Bar graph showing the effect of SMase on LDL aggregation (turbidimetry at 405 nm) (**B**) Gradient gel electrophoresis (GGE) and (**C**) Agarose gel electrophoresis showing LDL electrophoretic mobility of AgLDL in comparison to nLDL. Data are shown as mean ± SEM of three experiments performed in triplicate. Statistical significance was determined by Student’s *t*-test. O/N, overnight; LDL, low-density lipoprotein; nLDL, native LDL; AgLDL, aggregated LDL. Full uncropped blots can be found in Figure S5.

Turbidimetry assays (absorbance at 405 nm) showed that SMase strongly increased human LDL aggregation after 24 h (from 0.123 ± 0.26 to 1.21 ± 0.12, *p* < 0.001) (Figure 1A). Gradient gel electrophoresis (GGE) (Figure 1B) and agarose gels (Figure 1C) were used to visualize the modification of LDLs caused by SMase. In native GGE gels, the position of the LDL particle was determined by particle size. SMase caused a partial loss of the band corresponding to LDLs (Figure 1B). In Figure 1B, small LDL aggregates are marked with a red arrow while the lack of biggest LDL bands of AgLDL can be attributed to the fact that the pore size of GGE gels precludes the entrance of the largest LDL aggregates. In agarose gels (Figure 1C), where LDLs run according to its negative charge, AgLDL appeared as a diffuse band compared to the well-defined LDL band.

### 3.2. Effect of nLDLs and AgLDLs on Intracellular CE/FC Ratio in PANC-1, RWP-1 and Capan-1 Cells

**Figure 2:**
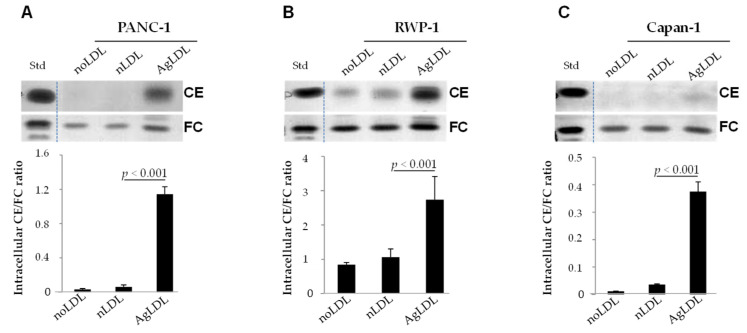
Effects of nLDLs and AgLDLs on the intracellular CE/FC ratio in PANC-1, RWP-1 and Capan-1 pancreatic tumor cells. Cells were untreated or treated with nLDL or AgLDL for 2 h. Representative thin layer chromatography (TLC) images show cholesteryl ester (CE) and free cholesterol (FC) bands; the bar graphs show the intracellular CE/FC ratio in PANC-1 (**A**), RWP-1 (**B**) and Capan-1 (**C**) cell types. Data are shown as the mean ± SEM of three experiments performed in triplicate. Statistical significance was determined by Student’s *t*-test. CE, cholesteryl ester; FC, free cholesterol; LDL, low-density lipoprotein; nLDLs, native LDLs; AgLDLs, aggregated LDL. Uncropped blots can be found in Figure S6.

**Figure 3:**
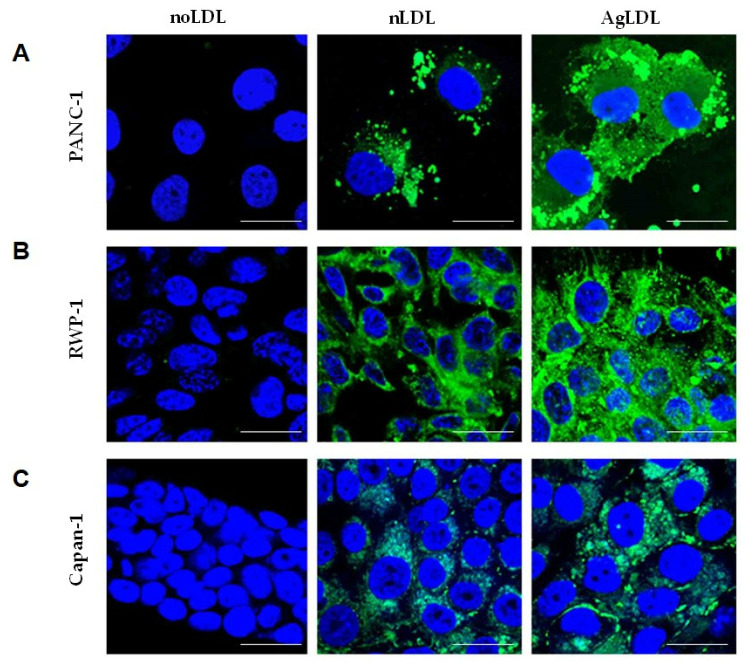
Effects of nLDLs and AgLDLs on lipid droplet formation in pancreatic tumor cell lines. Confocal laser microscopy images show BODIPY-stained lipid droplets (green dots) and nuclei (blue) in the (**A**) PANC-1, (**B**) RWP-1 and (**C**) Capan-1 cell lines. Scale bar, 25 μm. LDL, low-density lipoprotein; nLDL, native LDLs; AgLDL, aggregated LDLs.

We compared the effect of nLDLs and AgLDLs (100 µg/mL, 2 h) on the intracellular cholesteryl ester (CE)/free cholesterol (FC) ratio in the human pancreatic cancer cell lines PANC-1 (Figure 2A), RWP-1 (Figure 2B) and Capan-1 (Figure 2C) by thin layer chromatography (TLC). In the absence of LDL, PANC-1 and Capan-1 cells did not contain CE, while RWP-1 has a moderate CE content. At 2 h, nLDLs did not or only slightly increased the CE content of these cells lines; however, AgLDL strongly increased the CE/FC ratio in all pancreatic cancer cells (Figure 2). These results were corroborated by BODIPY staining and confocal microscopy, showing higher upregulatory effects of AgLDLs as compared to nLDLs on both size and abundance of lipid droplet (green dots) in PANC-1 (Figure 3A), RWP-1 (Figure 3B) and Capan-1 (Figure 3C) cell lines.

### 3.3. Effect of Peptide DP3 on SMase-Induced LDL Modification

**Figure 4:**
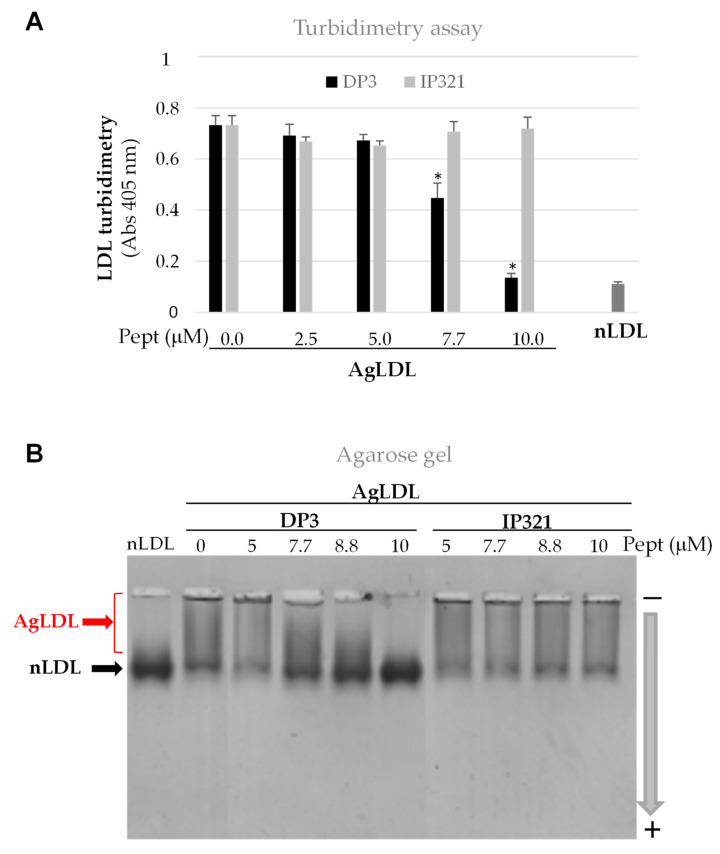
Effects of peptides DP3 and IP321 on SMase-induced LDL aggregation. LDLs were used at 1.44 mg/mL ApoB. The peptides DP3 and IP321 were tested at increasing concentrations (0, 2.5, 5, 7.7, 8.8 and 10 µM). The effects of peptides on LDL aggregation were monitored by measuring turbidimetry (**A**) and agarose gel electrophoretic mobility (**B**). Results are shown as mean ±SD of three independent experiments (n = 5 biological replicates). * *p* < 0.001, DP3 vs. IP321. Statistical significance was determined by Student’s *t*-test. LDL, low-density lipoprotein; nLDL, native LDLs; AgLDL, aggregated LDLs; pept: peptide. Detailed information can be found in Table S1 and Figure S7.

To study the effects of the peptides on SMase-induced LDL aggregation, LDL were incubated with SMase in the absence or presence of DP3 (anti-LDL aggregation peptide) or IP321 (negative control peptide), as previously detailed [29]. The effect of peptides on SMase-induced LDL aggregation was tested at several peptide concentrations (0, 2.5, 5, 7.7, 8.8 and 10 µM). Turbidimetry studies showed that DP3 significantly reduced SMase-LDL aggregation at 18 h in a dose-dependent manner (Figure 4A). As expected, the negative IP321 control peptide did not inhibit SMase-LDL aggregation, at any dose tested. In addition, agarose gels showed that the AgLDL band was more diffuse than the well-defined LDL band (Figure 4B). AgLDLs in the presence of DP3 (at concentrations higher than 5 µM) exhibited a similar band pattern as nLDLs. In contrast, IP321 did not change the running pattern of AgLDLs. We selected the concentration of 10 µM to perform further comparative studies with these peptides.

### 3.4. Effect of DP3 on Intracellular CE/FC Ratio Induced by AgLDL in PANC-1, RWP-1, and Capan-1 Pancreatic Tumor Cells

**Figure 5:**
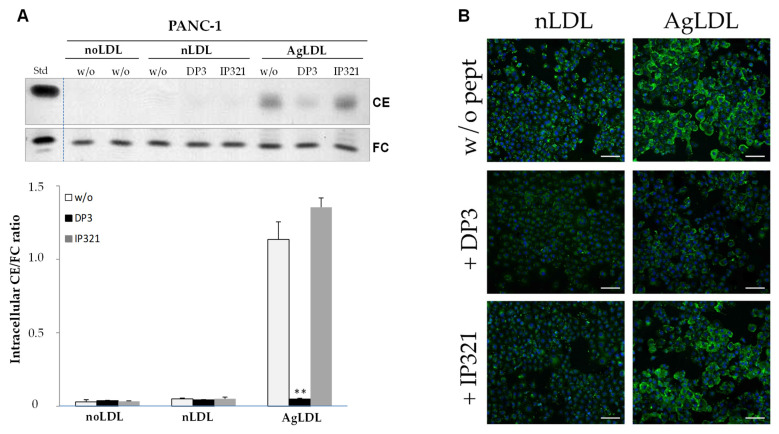
Effect of peptides DP3 and IP321 on the intracellular CE/FC ratio and lipid droplet formation induced by nLDLs and AgLDLs in PANC-1 cells. Cells were untreated (noLDL) or treated with nLDLs or AgLDLs in the absence (w/o peptide) or presence of DP3 or IP321 (10 μM, 2 h). (**A**) Representative thin layer chromatography (TLC) image showing CE and FC bands and bar graph showing the intracellular CE/FC ratio in PANC-1. Data are shown as the mean ± SEM of three experiments performed in triplicate. ** *p* < 0.001. Statistical significance was determined by Student’s *t* test. (**B**) Confocal laser microscopy images show BODIPY-stained lipid droplets (green dots) and nuclei (in blue). Scale bar, 100 μm. CE, cholesteryl esters; FC, free cholesterol; LDL, low-density lipoprotein; nLDL, native LDLs; AgLDLs, aggregated LDLs. Detailed information can be found in Figure S8.

Previous studies from our group have shown the high efficacy of LRP1-derived retro-enantio peptides in inhibiting LDL aggregation and vascular smooth muscle cell cholesterol loading [29,30]. Our current TLC results showed the high efficacy of DP3 in reducing intracellular CE/FC ratio induced by AgLDL in PANC-1 (Figure 5A). Similar results were observed in RWP-1 (Figures S1A and S9) and Capan-1 (Figures S1B and S9) cells. The control peptide IP321 did not decrease the intracellular CE/FC ratio caused by AgLDL in any of the cell types tested. As expected, DP3 or IP321 had no effect on the nLDL-induced intracellular CE/FC ratio in these cells. Confocal microscopy experiments (Figure 5B) confirmed that DP3, but not IP321, reduced lipid droplet formation in PANC-1 cells exposed to AgLDL (Figure 5B).

### 3.5. Time-Course Effect of nLDLs and AgLDLs on PANC-1 Cholesteryl Ester Loading and Proliferation

**Figure 6:**
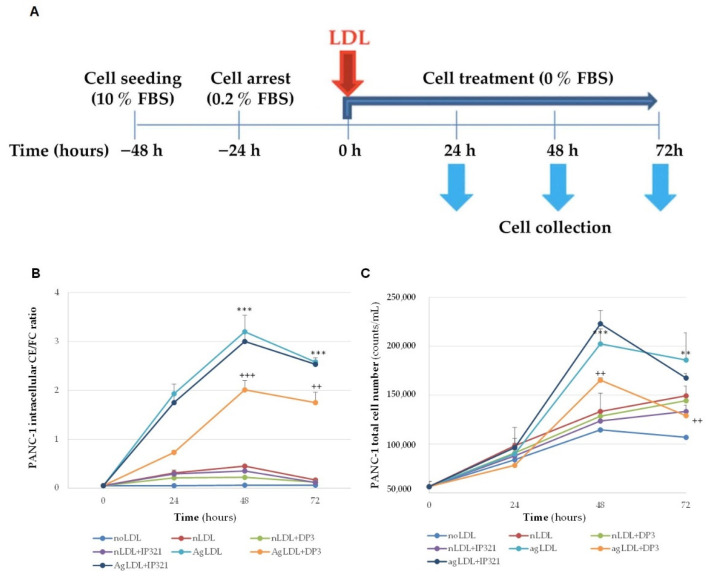
Time-course effects of nLDL and AgLDL on intracellular CE/FC ratio and PANC-1 total cell number. (**A**) Schematic representation of the experimental setting used to test the effect of LDL on intracellular CE/FC ratio and total cell number. Cells were untreated or treated with nLDL or AgLDL for increasing times (0, 24, 48, and 72 h) in the absence or presence of peptides (10 µM). (**B**) Line graphs showing the intracellular ratio between CE and FC quantified bands at 0, 24, 48 and 72 h of PANC-1 exposure to AgLDLs (**C**) Line graphs showing the flow cytometry quantification of total PANC-1 cell number after their exposure to nLDLs and AgLDLs for increasing times. Data are shown as the mean ± SD of two independent experiments (three to six biological replicates). ** *p* < 0.01, *** *p* < 0.001 (noLDL vs. AgLDL); ++ *p* < 0.01, +++ *p* < 0.001 (AgLDL + DP3 vs. AgLDL + IP321). Statistical significance was determined by Student’s *t* test. LDL, low-density lipoprotein; nLDL, native LDLs; AgLDLs, aggregated LDLs, CE cholesteryl esters; FC free cholesterol. Raw data can be found in File S1 and flow cytometry dot plots can be found in Figure S11.

To explore the potential of nLDL and AgLDL to induce PANC-1 proliferation, we analyzed the potential time-course effects of these lipoproteins on intracellular cholesterol accumulation and cell proliferation over time. In addition, peptides were used as an experimental strategy to determine the causality between inhibition of LDL aggregation-lipid droplet formation and cell growth blockade. We selected PANC-1 to perform cell growth studies because this cell line harbors the most frequent genetic alteration present in human PDAC (K-Ras and p53 mutations, CDKN2A loss) and is a well-recognized in vitro model for this pathology. The intracellular CE/FC ratio and cell number (total, live and dead cells) analyzed by flow cytometry were tested at different time points (0 h, 24 h, 48 h and 72 h) (Figure 6A and Figures S2, S3, and S10). Intracellular lipid studies evidenced that AgLDL strongly increased the intracellular CE/FC ratio at each tested time while nLDL showed no significant CE/FC increase along time (Figure 6B). DP3 (but not IP321) inhibited AgLDL-induced CE/FC ratio at all tested times. As expected, DP3 or IP321 did not alter CE/FC ratio in PANC-1 cells exposed to nLDL (Figure 6B).

To measure the net effect of lipoproteins on cell growth, cells were depleted of serum in the cell culture media for 72 h during the addition of LDLs (Figure 6A). Flow cytometry analysis was used to quantify total, live and dead cell numbers in each condition. AgLDLs significantly increased the total cell number after 48 h and 72 h of treatment in comparison to noLDL (Figure 6C and File S1). Of note, we found a slight reduction of total cell number from 48 to 72 h in PANC-1 exposed to AgLDL (Figure 6C) concomitantly with an increase of dead cells (Figure S4), likely due to cell stress caused by a mitogen stimulus (AgLDLs) in the absence of serum for a long time. Finally, we analyzed the effects of the inhibition of LDL aggregation in PANC-1 cell growth using the DP3 peptide. As shown in Figure 6C, DP3 significantly reduced the total cell number of AgLDL-exposed PANC-1 cells for 48 h and 72 h in comparison to cells exposed to AgLDL alone or AgLDL + IP321. The reducing effects of DP3 on the AgLDL-exposed PANC-1 cell number cannot be attributed to DP3 cytotoxic effects, as the dead cell number was similar in AgLDL + DP3 compared to w/o peptide or AgLDL + IP321 groups, either at 48 h or 72 h (Figure S4).

## 4. Discussion

During tumor evolution, both the content and organization of the ECM have been reported to be altered in close concert with the hypoxification and acidification of the tumors [39,40]. In this landscape, cholesterol and lipoproteins are essential for the metabolic adaptation in cancer, and lipid droplet phenotypes are crucial for chemoresistance and metastasis [41,42,43]. Acidic extracellular pH has been proposed to enhance the local availability of lipoprotein by increasing proteoglycan LDL retention and modification [11], as previously shown in the arterial intima in the context of atherosclerosis [10]. Results from the present study show that aggregated LDLs strongly upregulate the intracellular CE content of pancreatic tumor cells.

Aggregated LDL originated by the lipolytic effects of SMase on LDL particles might be relevant as a source of CE for pancreatic tumor cells, since SMase has been previously reported to be activated by acidic extracellular pH in metastatic melanomas [16]. Thus, it seems plausible that ECM proteoglycan-retained LDLs are rapidly aggregated by a permanently activated SMase status in the tumoral stroma. Our results now show that AgLDLs (generated by exposure of LDL to SMase) have much higher potential than native LDLs in increasing the intracellular CE/FC ratio and lipid droplet formation in all tested pancreatic tumor cells (PANC-1, RWP-1 and Capan-1), although the highest CE/FC ratio increase was observed in PANC-1 and RWP-1 cells. Our results show for the first time that LDL aggregation determines the intracellular CE/FC ratio and strongly favors the formation of lipid droplet phenotypes in pancreatic tumor cancer cells.

As previously shown in human vascular smooth muscle cells [29,30], results from the present study also show that DP3, which efficiently blocked SMase-induced LDL aggregation, also inhibited intracellular CE/FC ratio and lipid droplet formation in pancreatic tumor cells. These results demonstrate a close relationship between the inhibitory activity of peptides on SMase-induced LDL aggregation and PANC-1 loading and support the hypothesis that ECM-retained aggregated LDLs could be a source of cholesterol for tumors. In pancreatic cancer, the presence of lipid droplets is closely related with tumor aggressiveness; these droplets are formed by CEs from exogenous sources [24,25]. Moreover, PDAC tumors are enriched in activated-pathways associated with lipoprotein-related metabolism [26] and have a prominent stroma made up of ECM and abnormal blood vessels [12,13]. Therefore, it is plausible that ECM-retained ApoB100 LDLs could be a key factor for disease progression and be a target for new diagnostic and therapeutic approaches.

Here, we found that AgLDLs induced an increase in PANC-1 cell growth at 48 h and 72 h, which was impaired by DP3 but not by IP321, indicating the association between LDL aggregation/intracellular CE accumulation induced by AgLDLs and PANC-1 cell growth. Of note, PANC-1 cell growth was undetectable after 24 h in the presence of AgLDLs, even though a strong increase in the CE/FC ratio (caused by the modified LDLs) was evident. These results suggested that the induction of proliferation mediated by increased intracellular cholesterol (in the absence of FBS) requires longer than 24 h. Indeed, the PANC-1 population doubling time is approximately 32 h when cells exposed to complete medium containing 10% FBS, which is a strong mitogenic stimulus. This can also explain why AgLDL’s (or nLDL’s) effects on cell proliferation were not detectable until 48 h.

In PANC-1 cells, nLDLs that only slightly increased the intracellular CE/FC ratio, steadily increased cell growth, suggesting that other non-cholesterol-related mechanisms are involved on the induction of proliferation by nLDLs on PANC-1 cells. Previous studies have shown the upregulatory effects of LDLs on signal pathways and genes related to the proliferation of tumor cells; however, whether or not the effects depend on changes on the intracellular lipids had not yet been explored [44,45]. Patients with breast cancer and elevated LDL-cholesterol levels typically present with more aggressive cancers at advanced stages [46]. Elevated levels of circulating LDLs in patients with different types of cancer were related to a greater predisposition to the appearance of metastases in the lymph nodes; consequently, having high LDL levels can be a good indicator of cancer progression [47,48]. Previous studies have reported that the low-density lipoprotein receptor (LDLR), the cellular receptor of LDLs, is overexpressed in PDACs as compared to healthy pancreas [26], indicating the marked dependency of pancreatic tumor cells on cholesterol to proliferate. In addition, we and others have shown that intracellular CE levels are key determinants of proliferation and aggressiveness in breast cancer [49], leukemia [50], glioma [51] and prostate cancer [52].

Our results provide evidence that lipid reprogramming allows pancreatic cancer cells to use a new key pathway that supplies CEs to tumor cells. Importantly, recent data have shown that cholesterol-derived metabolites play a crucial role in tumor immune evasion, favoring the recruitment of immunosuppressive cells (neutrophils, myeloid derived suppressor cells and macrophages) and inhibiting immune-effector cells (CD8 T lymphocytes) [53]. Moreover, increased cholesterol loading and changes in lipid metabolism by vascular cells from the stroma can also modulate tumor angiogenesis [21,22], leading to altered abnormal vessels, which exacerbates the immunosuppression [23]. Consequently, considering the high proportion of stroma in PDAC, it is tempting to speculate that targeting LDLs/cholesterol loading in pancreatic cancer would have a strong impact on tumor cell behaviour.

The potential anti-tumoral relevance of peptides blocking LDL atherogenity should not go unnoticed. Previous studies have already shown that inhibition of the enzyme that catalyzes cholesteryl esterification, acyl coenzyme A: cholesterol acyltransferase (ACAT), suppresses growth and metastasis of pancreatic cancer in an orthotropic mouse model of pancreatic cancer [27,54]. By facilitating the increase of intracellular free cholesterol levels, highly cytotoxic for the cells, the inhibition of ACAT by avasimibe modulates tumor growth and metastasis in animal models. Results from the present study now show that anti-LDL aggregation peptides, such as DP3, efficiently inhibit the formation of intracellular CE lipid droplets that support tumor cell proliferation. A key advantage of these peptides might be that they could limit proliferation at very early stages of disease, which would be crucial for controlling tumor growth.

## 5. Conclusions

These results highlight the potential role of LDL aggregation and the subsequent increase of cholesterol uptake in the onset and progression of PDAC. Several key aspects must now be addressed, such as testing the relevance of this in vitro-tested mechanism in in vivo mouse models.

## Data Availability

Data supporting reported results can be found in the Supplementary Materials.

## Supplementary Materials

The following supporting information can be downloaded at: https://www.mdpi.com/article/10.3390/cancers14040890/s1, Figure S1: Effect of peptides DP3 and IP321 on the intracellular CE/FC ratio induced by nLDLs and AgLDLs in RWP-1 and Capan-1 cells, Figure S2: Time-course effects of nLDLs and AgLDLs on intracellular CE/FC ratio in PANC-1 cells, Figure S3: Optical microscopy of PANC-1 cells exposed to nLDL, and AgLDL ± DP3/IP321 peptides, Figure S4: Flow Cytometric analysis of cell proliferation and viability of PANC-1 cell cultures stained with PI, Figure S5: Uncropped blot for main Figure 1, Figure S6: Uncropped blots for main Figure 2, Figure S7: Uncropped blot for main Figure 4B, Figure S8: Uncropped blot for main Figure 5A, Figure S9: Uncropped blots for Figure S1, Figure S10: Uncropped blots for Figure S2, Figure S11: Flow cytometry dot plots for Figure 6C and Figure S4, Table S1: Raw data for Figure 4A, File S1: Flow Cytometry Analysis, raw data PANC-1 cell concentration (counts/mL) shown in Figure 6C, Figures S4 and S11.

## Abbreviations

ACAT acyl-coenzyme A: cholesterol acyltransferase; BSA bovine serum albumin; CAFs cancer-associated fibroblasts; CE cholesteryl esters; ECM extracellular matrix; FC free cholesterol; GGE Gradient gel electrophoresis; HPLC High Performance Liquid Chromatography; LD lipid-droplet; LDL Low-density lipoproteins; LDLR low-density lipoprotein receptor; LRP1 low density lipoprotein receptor-related protein 1; MALDI-TOF Matrix-Assisted Laser Desorption/Ionization-TOF; NEFA Non-Esterified Fatty Acids; nLDL native low-density lipoproteins; PBS phosphate buffered saline; PDAC Pancreatic ductal adenocarcinoma; PGs proteoglycans; SD standard deviation; SMase sphingomyelinase; TLC thin layer chromatography; VLDL very low density lipoprotein.
