# Supplementary material for: Peptides against Low Density Lipoprotein (LDL) Aggregation Inhibit Intracellular Cholesteryl Ester Loading and Proliferation of Pancreatic Tumor Cells
Source: Cancers (Basel). 2022 Feb 11;14(4):890. doi: 10.3390/cancers14040890 (PMC8869901; doi:10.3390/cancers14040890)
Supplement: Supplementary file 1 [file cancers-14-00890-s001.zip › cancers-1588998-supp/cancers-1588998-supplementary-final.pdf]

Supplementary Materials

Peptides against Low Density Lipoprotein (LDL) Aggregation Inhibit Intracellular Cholesteryl Ester Loading and Proliferation of Pancreatic Tumor Cells

Aleyda Benitez-Amaro, Neus Martínez-Bosch, Noemí Manero-Rupérez, Lene Claudi, Maria Teresa La Chica Lhoëst, Marta Soler, Lia Ros-Blanco, Pilar Navarro and Vicenta Llorente-Cortés

1. Supplementary Figures and Tables

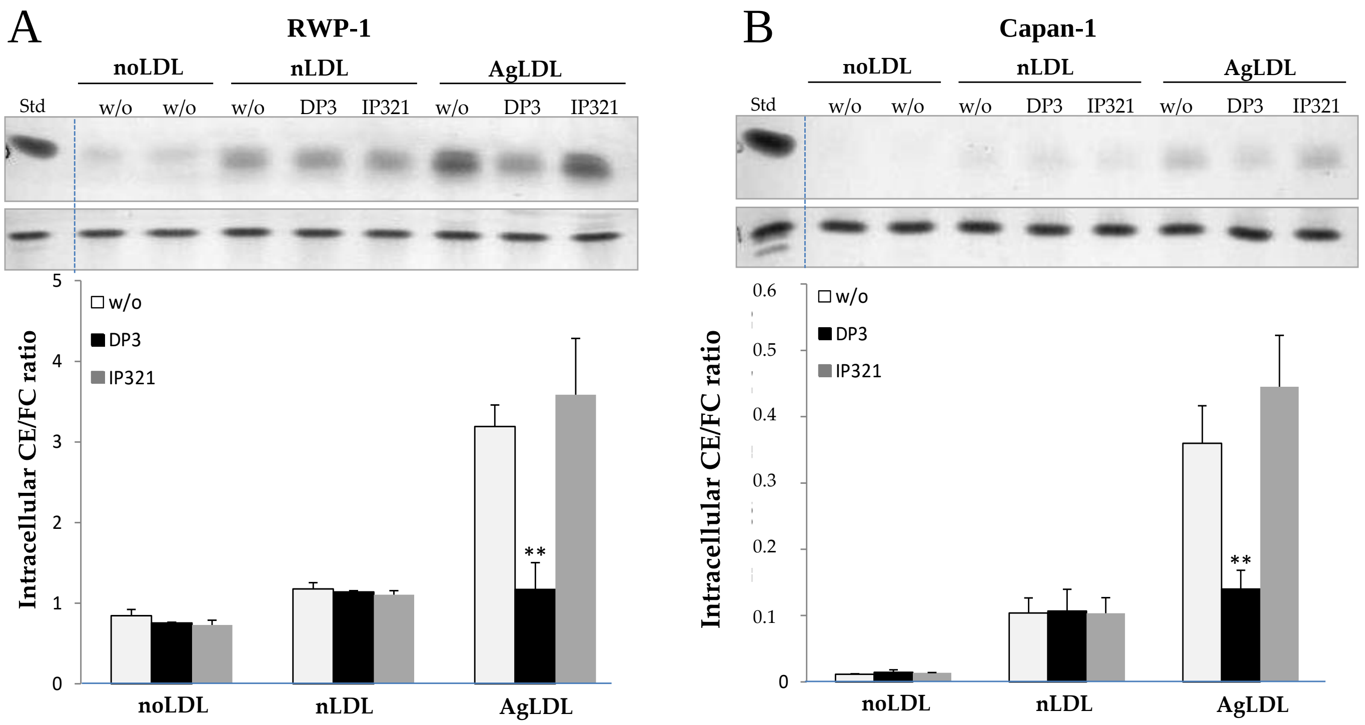

Figure S1. Effect of peptides DP3 and IP321 on the intracellular CE/FC ratio induced by nLDLs and AgLDLs in RWP-1 and Capan-1 cells. Cells were untreated or treated with nLDLs or AgLDLs in the absence (w/o peptide) or presence of DP3 or IP321 (10 μM, 2 h). Representative thin layer chromatography (TLC) image showing CE and FC bands and bar graph showing the intracellular CE/FC ratio in RWP-1 (**A**) and Capan-1 (**B**) tumor cells. Data are shown as the mean ± SEM, $n = 3$ experiments (performed by triplicate). ** $p < 0.001$ vs. w/o peptide or IP321. CE, cholesteryl esters; FC, free cholesterol; LDL, low-density lipoprotein; nLDL, native LDLs; AgLDL, aggregated LDLs; w/o, without. Full uncropped blots can be found in Figure S9.

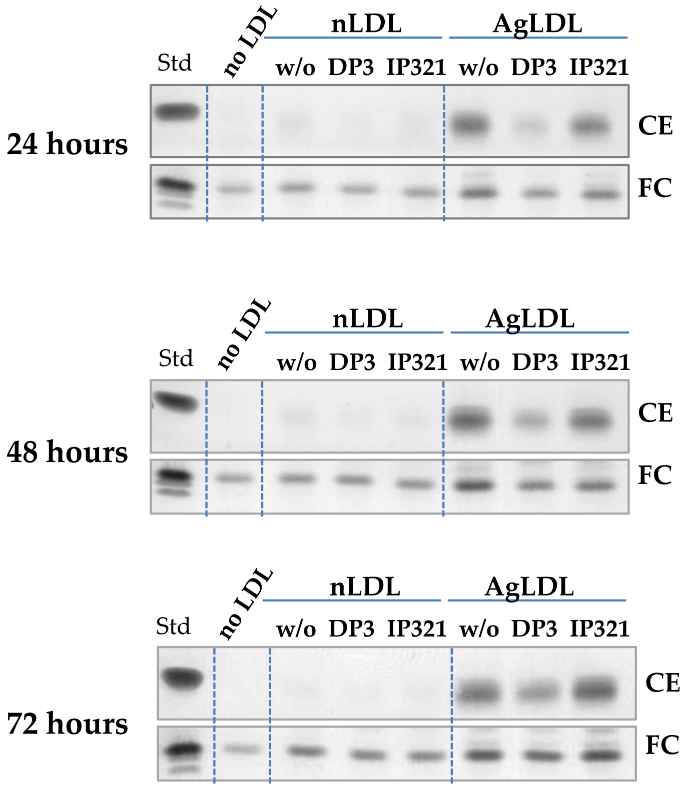

Figure S2. Time-course effect of nLDLs and AgLDLs on intracellular CE/FC ratio in PANC-1 cells. Cells were untreated or treated with nLDLs or AgLDLs for increasing times (0, 24, 48, and 72 h) in the absence or presence of peptides (10 μM). Representative thin layer chromatography (TLC) image showing CE and FC bands. CE, cholesteryl esters; FC, free cholesterol; LDL, low-density lipoprotein; nLDL, native LDLs; AgLDL, aggregated LDLs; Std, standards; w/o, without peptide. Full uncropped blots can be found in Figure S10.

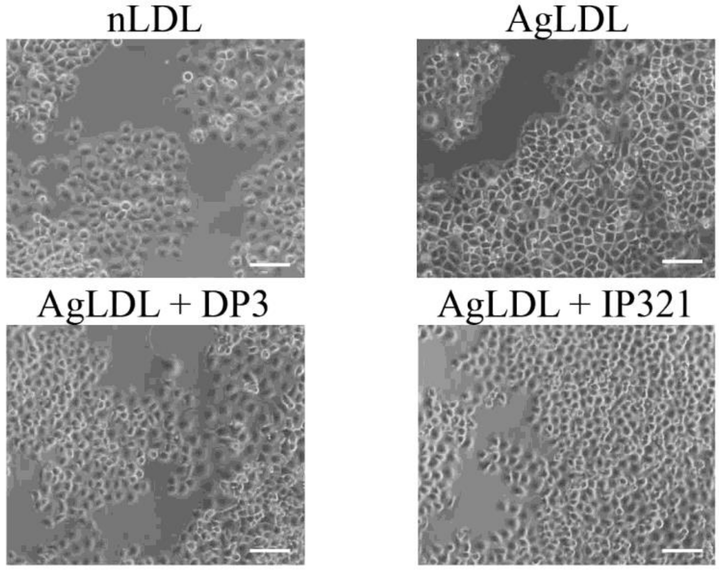

Figure S3. Optical microscopy representative images of PANC-1 cells exposed to nLDL, and AgLDL (1.44 mg/mL, 48 h) in the absence or presence of the DP3 or IP321 peptides. LDL, low-density lipoprotein; nLDL, native LDLs; AgLDL, aggregated LDLs. Scale bar, 100 μm.

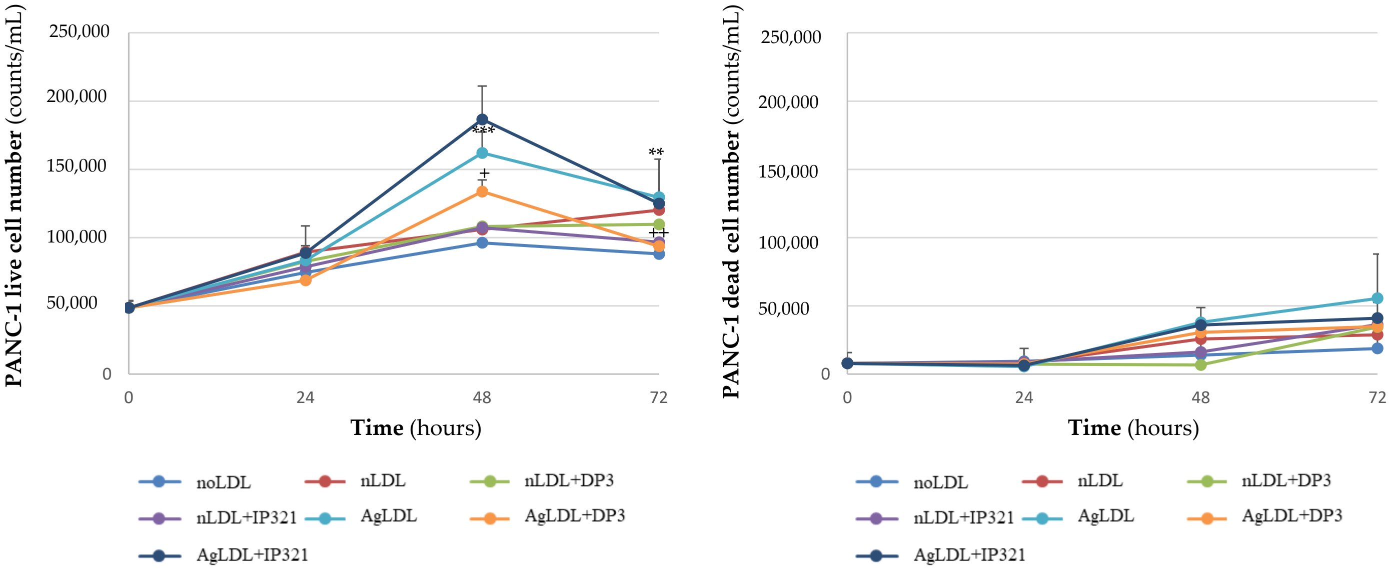

Figure S4. Flow Cytometric analysis of cell proliferation and viability of PANC-1 cell cultures stained with propidium iodide. Line graphs showing the progression of live (**left**) and dead (**right**) cells along time for each condition. Data are shown as the mean ± SD. $^{**}p < 0.01$, $^{***}p < 0.001$ (AgLDL vs. noLDL); $+p < 0.05$, $++p < 0.01$ (AgLDL + DP3 vs. AgLDL + IP321). For further comparisons please see Table S1. Statistical significance was determined by Student's *t* test. LDL, low-density lipoprotein; nLDL, native LDLs; AgLDL, aggregated LDLs. Raw data can be found in File S1.

A

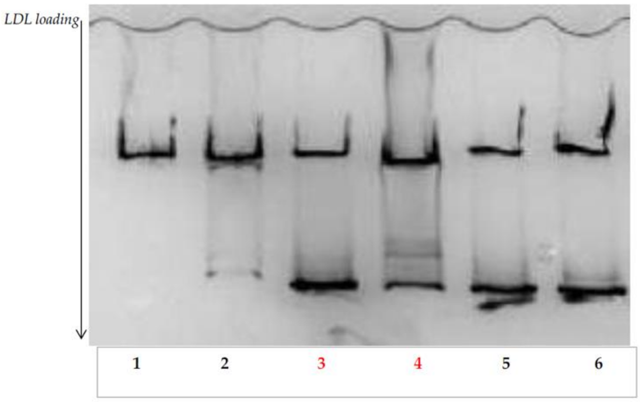

1	no LDL
2	AgLDL
**3**	**nLDL**
4	AgLDL
5	nLDL
6	nLDL

B

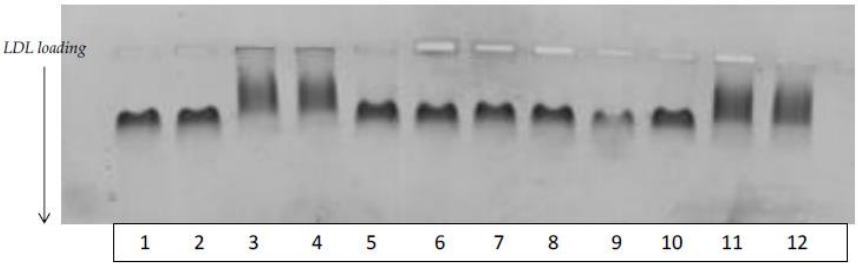

1	nLDL	
2	nLDL	
3	AgLDL	w/o pept
4	AgLDL	w/o pept
5	AgLDL	DP3
6	AgLDL	DP4
7	AgLDL	DP5
8	AgLDL	DP6
9	AgLDL	DP7
10	AgLDL	DP8
11	AgLDL	IP321
12	AgLDL	IP321

Figure S5. Uncropped blots for main Figure 1.(**A**), Uncropped blots for Figure 1B (Gradient Gel Electrophoresis (GGE)). Lanes 3,4 are included in main Figure 1. (**B**), Uncropped blots for Figure 1C (Agarose Gel). Lanes 1-4 are included in main figure F. Right, lane loading description. LDL, low-density lipoprotein; nLDL, native LDLs; AgLDLs, aggregated LDLs ; w/o pept, without peptide.

A

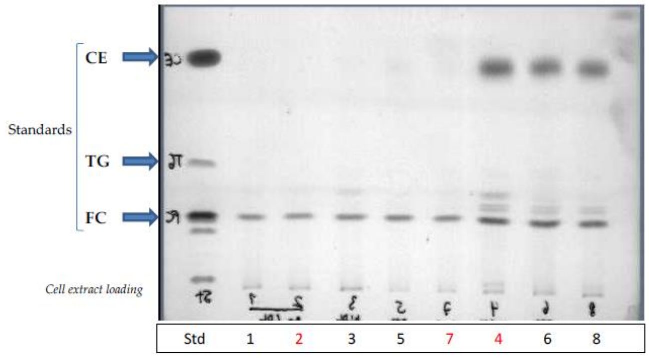

	Samples	FC	CE	CE/FC
1	noLDL	5.06	0.37	0.07
2	noLDL	4.75	0.05	0.01
3	nLDL	5.90	0.52	0.09
5	nLDL	6.07	0.97	0.16
7	nLDL	5.53	0.55	0.10
4	AgLDL	8.38	18.09	2.16
6	AgLDL	7.91	14.91	1.89
8	AgLDL	7.09	13.87	1.96

B

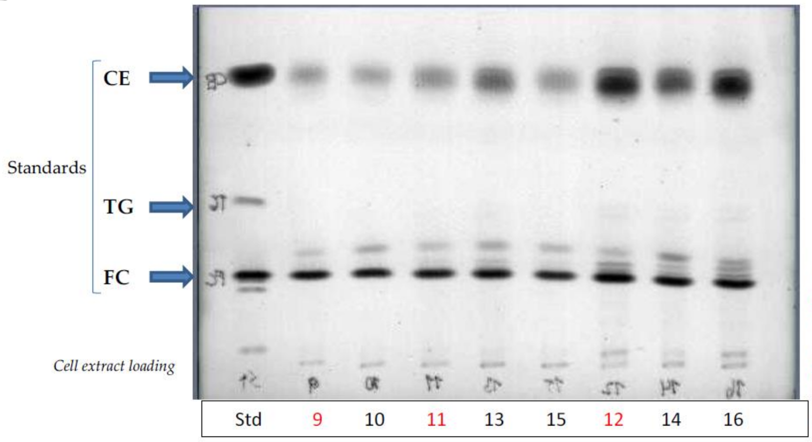

	samples	FC	CE	CE/FC
9	noLDL	4.52	4.09	0.91
10	noLDL	4.85	3.84	0.79
11	nLDL	4.56	5.12	1.12
13	nLDL+DP3	5.03	8.34	1.66
15	nLDL+IP321	4.45	4.79	1.08
12	AgLDL	4.30	14.54	3.38
14	AgLDL+DP3	4.40	9.50	2.16
16	AgLDL+IP321	3.48	14.20	4.09

C

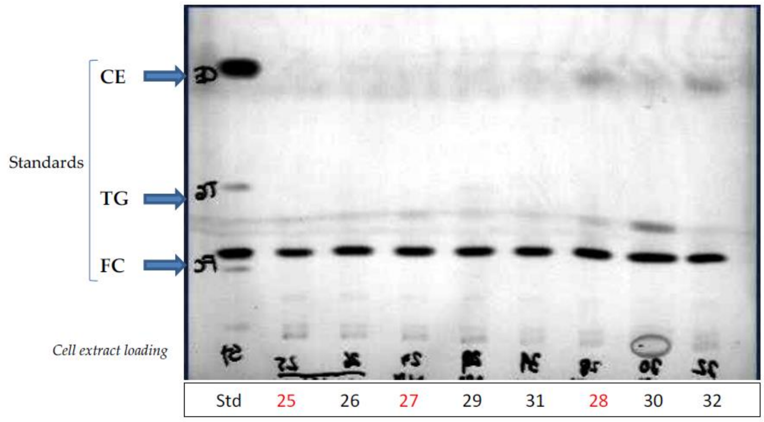

	samples	FC	CE	CE/FC
25	noLDL	8.56	0.15	0.02
26	noLDL	10.53	0.13	0.01
27	nLDL	11.08	0.42	0.04
29	nLDL+DP3	11.23	0.73	0.06
31	nLDL+IP321	10.66	0.94	0.09
28	AgLDL	11.94	3.87	0.32
30	AgLDL+DP3	12.51	2.02	0.16
32	AgLDL+IP321	10.15	5.10	0.50

Figure S6. Uncropped blots for main Figure 2. (**A**) Uncropped blot for Figure 2A, thin Layer Chromatography (TLC) (PANC-1 cells). Lanes 2,4,7 are included in the main Figure 2A. (**B**) Uncropped blot for Figure 2B, thin Layer Chromatography (TLC) (RWP-1). Lanes 9,11,12 are included in main Figure 2B. (**C**) Uncropped blot for Figure 2A, thin Layer Chromatography (TLC) (Capan-1 cells). Lanes 25,27,28 are included in main Figure 2C. Right, densitometry readings. LDL, low-density lipoproteins; nLDL, native LDLs; AgLDLs, aggregated LDLs ; w/o pept, without peptide; CE, cholesteryl esters; FC, free cholesterol; TG, triglycerides.

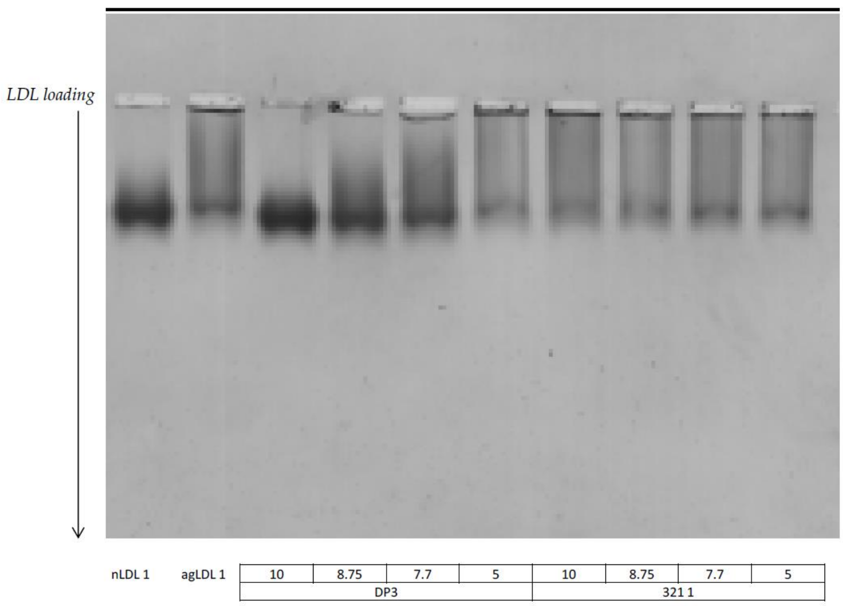

Figure S7. Uncropped blot for main Figure 4B. Whole agarose gel is included in the main figure. LDL, low-density lipoprotein; nLDL, native LDL; AgLDL, aggregated LDL.

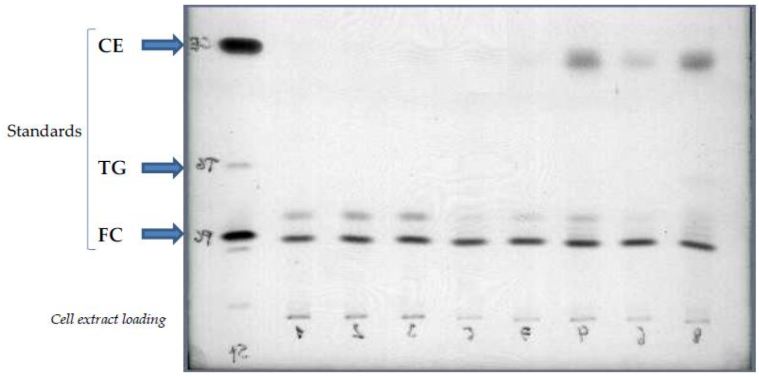

	samples	FC	CE	CE/FC
1	noLDL	7.56	0.03	0.00396925
2	noLDL	9.08	0.20	0.02250946
3	nLDL	9.25	0.50	0.05377445
5	nLDL+DP3	8.83	3.74	0.42392896
7	nLDL+IP321	8.98	0.53	0.0589211
4	AgLDL	10.01	10.51	1.05026111
6	AgLDL+DP3	9.00	2.97	0.33008602
8	AgLDL+IP321	8.17	10.69	1.30840498

Figure S8. Uncropped blot for main Figure 5A. Whole Thin Layer Chromatography (TLC) for PANC-1 cells is included in the main figure. Right, densitometry readings. LDL, low-density lipoproteins; nLDL, native LDLs; AgLDLs, aggregated LDLs ; CE, cholesteryl esters; FC, free cholesterol; TG, triglycerides.

A

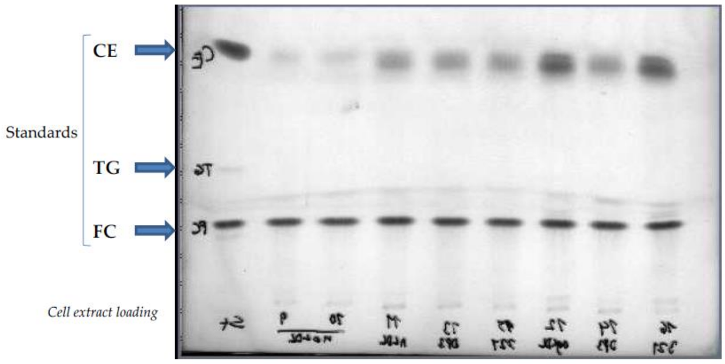

	samples	FC	CE	CE/FC
9	noLDL	4.52	4.09	0.90509512
10	noLDL	4.85	3.84	0.79147473
11	nLDL	4.56	5.12	1.12236805
13	nLDL+DP3	5.03	6.34	1.26090402
15	nLDL+IP321	4.45	4.79	1.07771257
12	AgLDL	4.30	14.54	3.38128735
14	AgLDL+DP3	4.40	6.22	1.413361
16	AgLDL+IP321	3.48	14.20	4.08729668

B

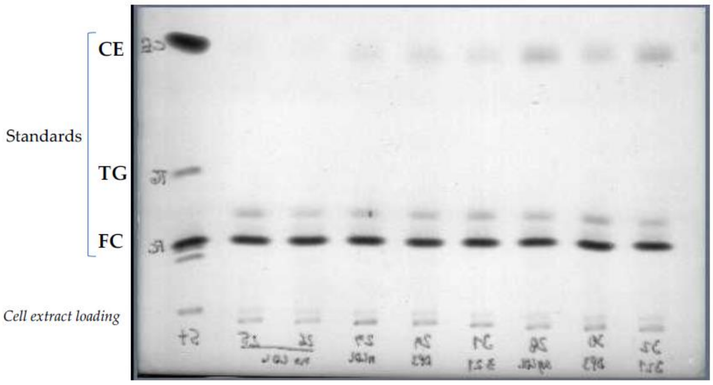

	samples	FC	CE	CE/FC
25	noLDL	10.31	0.01	0.00097034
26	noLDL	11.08	0.13	0.01190905
27	nLDL	11.05	1.43	0.12939964
29	nLDL+DP3	10.15	1.75	0.17227161
31	nLDL+IP321	10.58	1.36	0.12849417
28	AgLDL	10.53	4.18	0.39713257
30	AgLDL+DP3	10.86	2.20	0.20262769
32	AgLDL+IP321	10.34	4.05	0.39176841

Figure S9. Uncropped blots for Figure S1. (**A**): Uncropped blot for Figure S1A: Thin Layer Chromatography (TLC) (RWP-1 cells). Whole TLC is included in the the Figure S1. (**B**) Uncropped blot for Figure S1B: Thin Layer Chromatography (TLC) (Capan-1 cells). Whole TLC is included in the Figure S1. Right, densitometry readings. LDL, low-density lipoproteins; nLDL, native LDLs; AgLDLs, aggregated LDLs ; CE, cholesteryl esters; FC, free cholesterol; TG, triglycerides.

A

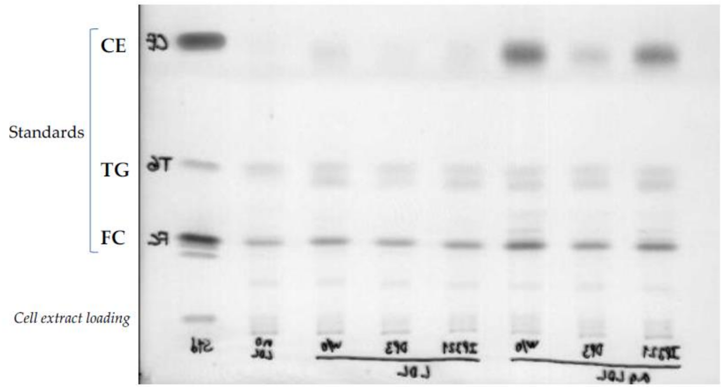

	samples	FC	CE	CE/FC
1	no LDL	5.09	0.18	0.03622871
2	LDL	7.11	2.50	0.35218799
3	LDL+DP3	6.10	0.79	0.12918444
4	LDL+IP321	6.03	1.49	0.24703988
5	AgLDL	11.59	20.63	1.78078301
6	AgLDL+DP3	7.55	5.29	0.6998504
7	AgLDL+IP321	9.34	16.30	1.74570423

B

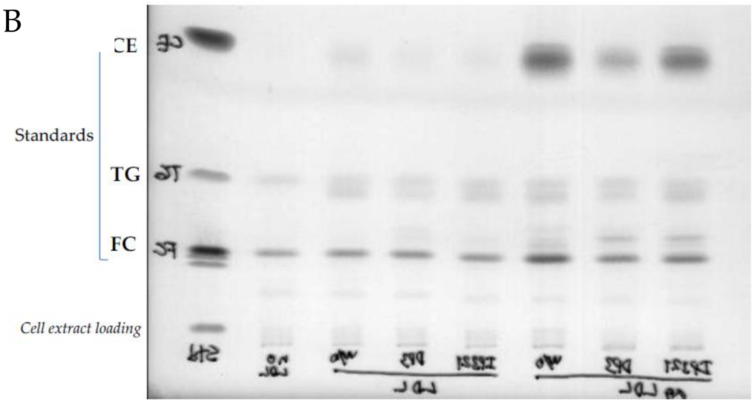

	samples	FC	CE	CE/FC
1	no LDL	4.16	0.14	0.03365605
2	LDL	5.59	2.52	0.4505776
3	LDL+DP3	5.38	1.29	0.23960785
4	LDL+IP321	5.56	1.66	0.29874793
5	agLDL	9.14	29.15	3.18857897
6	agLDL+DP3	6.54	9.07	1.38782886
7	agLDL+IP321	7.27	28.82	3.96174877

C

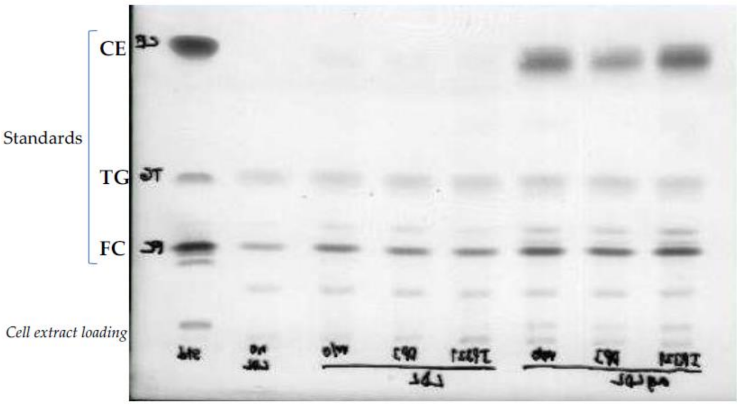

	samples	FC	CE	CE/FC
1	no LDL	2.51	0.20	0.08
2	nLDL	5.15	0.53	0.10
3	nLDL P3	4.31	0.32	0.07
4	nLDL 321	4.33	0.42	0.10
5	agLDL	7.46	16.75	2.24
6	agLDL P3	5.97	9.73	1.63
7	agLDL 321	6.19	17.68	2.85

Figure S10. Uncropped blot for Figure S2. (**A**) Thin Layer Chromatography (TLC) (PANC-1 cells 24 h). (**B**) Thin Layer Chromatography (TLC) (PANC-1 cells 48 h). (**C**) Thin Layer Chromatography (TLC) (PANC-1 cells 72 h). Whole TLC are included in Figure S2. Right, densitometry analysis. LDL, low-density lipoproteins; nLDL, native LDLs; AgLDLs, aggregated LDLs ; CE, cholesteryl esters; FC, free cholesterol; TG, triglycerides.

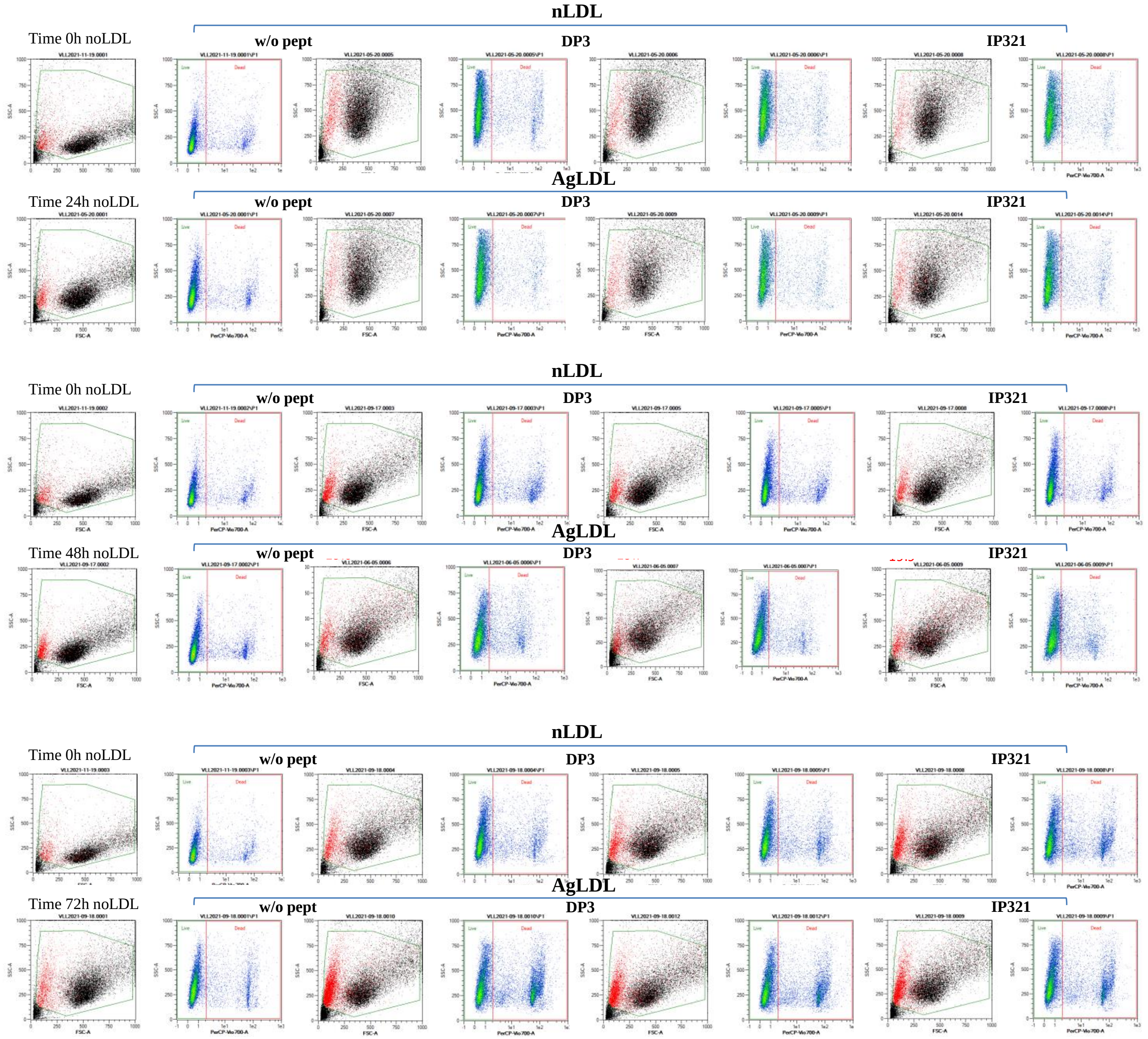

Figure S11. Flow Cytometry dot plots for Figure 6C and Figure S4. LDL, low-density lipoproteins; nLDL, native LDLs; AgLDLs, aggregated LDLs ; CE, cholesteryl esters; FC, free cholesterol; TG, triglycerides.

Table S1. Raw data for Figure 4A.

Details	Without Peptide	DP3 (2.5 µM)	DP3 (5 µM)	DP3 (7.7 µM)	DP3 (10 µM)	IP321 (2.5µM)	IP321 (5 µM)	IP321 (7.7 µM)	IP321 (10 µM)	nLDL
-	0.736	0.744	0.701	0.376	0.14	0.674	0.67	0.776	0.729	0.1
	0.78	0.647	0.647	0.512	0.149	0.65	0.623	0.691	0.782	0.104
	0.754	0.645	0.653	0.416	0.154	0.696	0.66	0.694	0.675	0.115
	0.703	0.722	0.69	0.428	0.119	0.657	0.652	0.687	0.732	0.117
	0.687	0.7	0.672	0.504	0.12	0.667	0.659	0.688	0.677	0.119
**Mean**	0.732	0.692	0.673	0.447	0.136	0.669	0.653	0.707	0.719	0.111
**SD**	0.0376	0.044	0.023	0.059	0.016	0.018	0.018	0.039	0.044	0.009

File S1 is attached as an independent Excel file that contains flow cytometry analysis, and raw data PANC-1 cell concentration (counts/mL) shown in Figure 6C, S4 and S11.
